# Prognostic Value of N-Terminal Pro-B-Type Natriuretic Peptide and High-Sensitivity C-Reactive Protein in Patients With Previous Myocardial Infarction

**DOI:** 10.3389/fcvm.2022.797297

**Published:** 2022-02-24

**Authors:** Ye-Xuan Cao, Sha Li, Hui-Hui Liu, Meng Zhang, Yuan-Lin Guo, Na-Qiong Wu, Cheng-Gang Zhu, Qian Dong, Jing Sun, Ke-Fei Dou, Jian-Jun Li

**Affiliations:** ^1^Cardiovascular Metabolism Center, State Key Laboratory of Cardiovascular Disease, Fu Wai Hospital, National Center for Cardiovascular Disease, Chinese Academy of Medical Sciences and Peking Union Medical College, Beijing, China; ^2^Department of Cardiology, Beijing Chaoyang Hospital Affiliated to Capital University of Medical Science, Beijing, China

**Keywords:** myocardial infarction, NT-proBNP, hs-CRP, prognosis, cardiovascular events

## Abstract

**Background:**

Patients with previous myocardial infarction (MI) have a poor prognosis and stratification for recurrent major adverse cardiovascular events (MACE) among these patients is of considerable interest. N-terminal pro-B-type natriuretic peptide (NT-proBNP) and high-sensitivity C-reactive protein (hs-CRP) are considered to be potential cardiovascular risk factors, but less is known about their prognostic importance in post-MI patients. This study aimed to evaluate the prognostic value of NT-proBNP and hs-CRP alone or together in patients who reported a prior MI.

**Methods:**

In this prospective study, we consecutively enrolled 3,306 post-MI patients to assess the recurrent MACE. The predictive values of NT-proBNP and hs-CRP alone and together were assessed by multivariable Cox regression using hazard ratios (HR) and 95% confidence intervals (CI).

**Results:**

During the 4-year follow-up period, 335 patients developed recurrent MACE. Multivariate Cox regression analysis showed a significant correlation between NT-proBNP levels and MACE (HR: 2.99, 95%CI: 2.06–4.36, *p* < 0.001), hard endpoints (HR: 5.44, 95%CI: 2.99–9.90, *p* < 0.001), cardiac mortality (HR: 5.92, 95%CI: 2.34–14.96, *p* < 0.001) and all-cause mortality (HR: 5.03, 95%CI: 2.51–10.09, *p* < 0.001). However, hs-CRP was not an independent predictor after adjusting for NT-proBNP. When patients were divided into six groups by using tertiles values of NT-proBNP and median values of hsCRP, patients with high NT-proBNP/hs-CRP values were 3.27 times more likely to experience MACE than patients with low NT-proBNP/hs-CRP values. The addition of NT-proBNP and hs-CRP to a prognostic model revealed a significant improvement in C-statistic, net reclassification, and integrated discrimination.

**Conclusions:**

Increased NT-proBNP levels were associated with long-term worse outcomes and the combination of NT-proBNP and hs-CRP has an incremental value in the further risk stratification of post-MI patients.

## Introduction

Despite the development of effective preventive measures, coronary artery disease (CAD) remains a leading cause of morbidity and mortality worldwide currently (1). Studies on patients with myocardial infarction (MI) have revealed that post-MI patients are at increased risk for recurrent events after revascularization and well-established pharmacological therapies, resulting in consequent reduction of long-term survival in this population (2). Therefore, management of clinical events in post-MI patients as secondary prevention remains a challenge for most cardiologists and appropriate risk stratification of these patients is considerable important for intensive secondary preventive strategies.

Recent research has concentrated on identifying biomarkers which could be used to identify high-risk population and reliably predict prognosis (3). In this regard, N-terminal pro-B-type natriuretic peptide (NT-proBNP) and high-sensitivity C-reactive protein (hs-CRP) are of considerable interest. NT-proBNP is considered a good indicator of left ventricle impaired function, which is caused by myocardial necrosis or ischemic dysfunction (4). Recent studies have demonstrated that elevated NT-proBNP concentration is secreted from hypoxic myocardium, even in the absence of left ventricular dysfunction (5, 6). Although NT-proBNP is the preferred biomarker for heart failure (HF), a number of studies have suggested that NT-proBNP is also a risk marker to predict major adverse cardiovascular events (MACE) in the general population and in patients with CAD (7–9). Despite the evidence, in clinical practice, the use of NT-proBNP is not yet consolidated in the risk assessment of patients with previous MI.

Inflammation plays an important role in the development, propagation and destabilizing plaques in the coronary arteries (10). Among the biomarkers, hs-CRP has become a widely-used laboratory indicator of inflammation (11). Numerous studies have demonstrated the independent prognostic relevance of hs-CRP for the risk of cardiovascular events in patients with CAD and also in apparently healthy population (12–14). Recently, a large randomized controlled trial found that canakinumab significantly reduced hs-CRP levels and recurrent MACE (15). In PCSK9 trials, even in patients with extremely low levels of low-density lipoprotein cholesterol (LDL-C), there was a stepwise risk increment according to the values of hs-CRP (16). However, the association between increased hs-CRP levels and long-term recurrent MACE in the post-MI patients has not been fully evaluated and there appears to be conflicting data (17), which requires further research.

In patients with CAD, a multi-marker approach to risk stratification has allowed a powerful short- and long-term prediction of heightened risk of MACE (18–21). Although information available indicates that NT-proBNP and hs-CRP might provide unique prognostic information in patients with previous MI, no study so far has assessed whether combined use of these biomarkers could improve the risk stratification.

Given that data regarding the prognostic value of NT-proBNP and hs-CRP in patients with prior MI are sparse, we assessed the independent prognostic utility of NT-proBNP and hs-CRP. The combined analysis of NT-proBNP and hs-CRP was also carried out to evaluate a multi-marker strategy in the individualization of risk stratification. These data may provide novel approach for predicting the prognosis of post-MI patients.

## Materials and Methods

### Study Design and Populations

In this observational study with a prospective cohort design, a total of 3,306 patients with history of MI, who were hospitalized in Fuwai Hospital, were consecutively collected from March 2009 to January 2019. ST-segment elevation MI (STEMI) was defined as the presence of persistent chest pain, new or presumed new ST-segment elevation in 2 or more contiguous leads and elevated cardiac biomarkers. Non-STEMI (NSTEMI) was diagnosed by ischemic symptoms and a positive cardiac biomarkers without new ST-segment elevation. We excluded subjects who met any of the following criteria: (1) patients aged <18; (2) had familial hypercholesterolemia; (3) had the presence of the typical symptoms of HF and a left ventricular ejection fraction (LVEF) <45%; (4) thyroid dysfunction; (5) severe liver and/or renal insufficiency or malignant disease.

This study complied with the Declaration of Helsinki and was approved by the hospital's ethical review board (Fu Wai Hospital & National Center for Cardiovascular Diseases, Beijing, China). Each participant provided written, informed consent before enrollment.

### Baseline Data Collection

Clinical data of each participant, including age, height, weight and blood pressure were obtained by experienced physicians and nurses when first enrolled. Hypertension was defined as a self-reported hypertension, currently taking anti-hypertensive drugs or recorded systolic blood pressure (SBP) ≥140 mmHg and/or diastolic blood pressure (DBP) ≥90 mmHg for three or more consecutive times. Participants with a fasting plasma glucose (FPG) level ≥7.0 mmol/L, 2-h serum glucose of the oral glucose tolerance test ≥11.1 mmol/L or currently using hypoglycemic drugs or insulin were defined as having diabetes. Body Mass Index (BMI) was calculated as weight in kilograms divided by the height in meters squared (kg/m^2^). Smokers were defined as subjects who consumed tobacco products within the past 6 months. The severity of coronary artery stenosis was evaluated by the Gensini score, which was scored according to stenosis position and severity (22).

Fasting blood samples (12 h overnight) were obtained between 7:00 and 8:00 a.m. from each participant. The concentrations of total cholesterol (TC), triglyceride (TG), LDL-C, high-density lipoprotein cholesterol (HDL-C), apolipoprotein A (apoA) and apolipoprotein B (apoB) were measured using an automatic biochemistry analyzer (Hitachi 7150, Japan). Plasma NT-proBNP concentration was measured with an electrochemiluminescence immunoassay (ECLIA) method (Roche, Germany) by a Roche modular analytics E170 immunoassay analyzer. Hs-CRP level was examined by immunoturbidimetry (Beckmann Assay 360, Bera, California). Other related biochemical and hematological indicators were measured according to standard tests.

### Follow-Up and Endpoints Assignment

Patients were followed up semi-annually by clinic revisit, detailed questionnaires sent by mail or telephone conducted by trained nurses or doctors. All events were carefully checked and verified by two experienced clinical physicians. The primary endpoint was MACE, which was a composite of newly developed MI, ischemic stroke, coronary revascularization and cardiac mortality. The three secondary endpoints included: (1) the hard endpoint was the combined endpoints of MI, ischemic stroke and cardiac mortality; (2) cardiac mortality; (3) all-cause mortality. The diagnosis was based on the International Statistical Classification of Diseases and Related Health Problems, 10th revision (ICD-10) codes. MI and ischemic stroke was determined based on the recording of ICD-10 code I21 and I63/I64, respectively. Cardiac mortality referred to ICD-10 code I00-I10, I11, I13, I20-I51, I70, and I74. Revascularization was defined as percutaneous coronary intervention (PCI) or coronary artery bypass grafting (CABG) beyond 3 months after discharged. Diagnosis of mortality was confirmed by hospital records, death certificates and information provided by family members. During the follow-up period, 15 patients were lost to follow-up. Finally, a total of 3,306 patients completed the follow-up and were included in the present study (Supplementary Figure S1).

### Statistical Analysis

The values were expressed as the mean ± standard deviation (SD) or median (Q1–Q3 quartiles) for the continuous variables and the number (percentage) for the categorical variables. Differences of continuous variables between groups were determined using the student's *t*-test, one-way ANOVA analysis, Mann–Whitney *U* test or Kruskal-Wallis H test where appropriate. Event rates were estimated using the Log-rank test and Kaplan-Meier (KM) analysis. Univariate and multivariate Cox proportional hazard models were used to estimate the relative risk of NT-proBNP or hs-CRP for each of the study end points using hazard ratios (HRs) with their 95% confidence intervals (CIs). Skewed variables were log transformed to achieve approximately symmetrical distributions. We performed several subgroup and sensitivity analyses to test the robustness of our findings. To assess the incremental value of adding continuous NT-proBNP and hs-CRP variable to established cardiovascular risk factors prediction model, we calculated Harrell's C-statistic, the continuous net reclassification improvement (NRI), and integrated discrimination improvement (IDI). A *P*-value <0.05 was considered statistically significant. Statistical analyses were performed using SPSS software (SPSS Inc., Chicago, Illinois) and R version 3.5.2 (Feather Spray).

## Results

### Patient Characteristics

The baseline characteristics of patients with or without recurrent MACE were described in Table 1. Patients with MACE were older and had lower LVEF (both *p* < 0.05). Additionally, the event group had a higher proportion of hypertension and diabetes, and higher levels of TC, LDL-C, HbA1c, hsCRP, and NT-proBNP compared with non-event group (all *p* < 0.05).

**Table 1:** Baseline characteristics of study patients.

**Variables**	**Total (*N* = 3,306)**	**Non-MACE (*N* = 2,971)**	**MACE (*N* = 335)**	***p*-value**
Age, y	61.2 ± 15.8	60.4 ± 15.7	68.5 ± 15.1	<0.001
Male, *n* (%)	2,644 (80.0)	2,395 (80.6)	249 (74.3)	0.060
BMI, kg/(m^2^)	24.32 ± 3.03	24.31 ± 3.04	24.44 ± 3.00	0.458
Family history of CAD, *n* (%)	408 (12.3)	362 (12.2)	46 (13.7)	0.414
Currently smoker, *n* (%)	1,992 (60.3)	1,796 (60.5)	196 (58.5)	0.491
Hypertension, *n* (%)	1,992 (60.3)	1,769 (59.5)	223 (66.6)	0.013
Diabetes, *n* (%)	1,161 (35.1)	1,016 (34.2)	145 (43.3)	0.001
Prior revascularization, *n* (%)	1,233 (37.3)	1,119 (37.7)	114 (34.0)	0.192
STEMI, *n* (%)	1,893 (57.3)	1,707 (57.5)	186 (55.5)	0.489
LVEF,%	59.90 ± 6.97	60.04 ± 6.98	58.64 ± 6.79	0.001
SBP, mmHg	125.28 ± 17.75	124.96 ± 17.68	128.09 ± 18.12	0.002
DBP, mmHg	75.68 ± 11.08	75.74 ± 11.10	75.11 ± 10.83	0.321
Gensini score	40 (16–70.5)	40 (16–70)	42 (14–86)	0.163
Total cholesterol, mmol/L	3.93 ± 0.98	3.91 ± 0.99	4.04 ± 0.92	0.022
HDL-C, mmol/L	1.02 ± 0.28	1.02 ± 0.28	1.00 ± 0.28	0.355
LDL-C, mmol/L	2.36 ± 0.83	2.35 ± 0.84	2.46 ± 0.79	0.024
Triglyceride, mmol/L	1.61 ± 0.84	1.61 ± 0.84	1.61 ± 0.80	0.958
Apolipoprotein A, mg/dL	1.26 ± 0.27	1.24 ± 0.28	1.26 ± 0.26	0.165
Apolipoprotein B, mg/dL	0.86 ± 0.32	0.85 ± 0.33	0.89 ± 0.27	0.083
Hs-CRP, mg/L	1.99 (0.95–5.11)	1.91 (0.94–4.81)	2.88 (1.16–9.59)	0.013
FPG, mmol/L	6.07 ± 1.98	6.05 ± 1.93	6.23 ± 2.36	0.118
HBA1C, %	6.42 ± 1.22	6.40 ± 1.21	6.59 ± 1.25	0.009
NT-proBNP, pg/mL	545.5 (175.1–1121.7)	509.6 (153.3–1053.1)	1070.3 (513.4–1507.0)	<0.001
Creatinine, umol/L	84.73 ± 24.71	84.59 ± 24.46	85.52 ± 27.01	0.514
Medication use
ACEI/ARB, *n* (%)	1,191 (36.0)	1,062 (35.7)	129 (38.5)	0.318
Beta-blockers, *n* (%)	1,739 (52.6)	1,556 (52.4)	183 (54.6)	0.434
Aspirin, *n* (%)	1,739 (52.6)	1,556 (52.4)	183 (54.6)	0.434
Statins, *n* (%)	2,366 (71.6)	2,141 (72.1)	225 (67.2)	0.061
CCB, *n* (%)	547 (16.5)	492 (16.6)	55 (16.4)	0.947
Follow-up medications
ACEI/ARB, *n* (%)	1,522 (46.0)	1,360 (45.8)	162 (46.3)	0.725
Beta-blockers, *n* (%)	2,572 (77.8)	2,320 (78.1)	252(75.2)	0.281
Aspirin, *n* (%)	3,196 (96.7)	2,877 (96.8)	319 (95.2)	0.834
Statins, *n* (%)	3,110 (94.1)	2,800 (94.2)	310 (92.5)	0.061
CCB, *n* (%)	1,269 (38.4)	1,145 (38.6)	124 (37.0)	0.947

*Data are expressed as the mean value ± standard deviation, median with 25th and 75th or number (%). MACE, major adverse cardiovascular events; BMI, Body mass index; CAD, coronary artery disease; STEMI, ST-segment elevation myocardial infarction; LVEF, left ventricular ejection fraction; SBP, systolic blood pressure; DBP, diastolic blood pressure; HDL-C, high-density lipoprotein cholesterol; LDL-C, low-density lipoprotein cholesterol; Lp(a), lipoprotein(a); hs-CRP, high sensitivity C-reactive protein; FPG, fasting plasma glucose; HbA1b, Hemoglobin A1c; NT-proBNP, N-terminal pro-brain natriuretic peptide; ACEI, angiotensin-converting enzyme inhibitor; ARB, angiotensin receptor blocker; CCB, calcium channel blockers*.

### NT-proBNP and Clinical Outcomes

Patients were divided into 3 groups according to plasma NT-proBNP levels (Supplementary Table S1). Patients with lower NT-proBNP levels were younger and more likely to be males while those in tertile 3 of NT-proBNP had higher prevalence of hypertension and diabetes and lower LVEF. There was an ascending gradient regarding the concentrations of TC, apoA, FPG, HbA1c and hsCRP across NT-proBNP tertiles.

**Figure 1:**
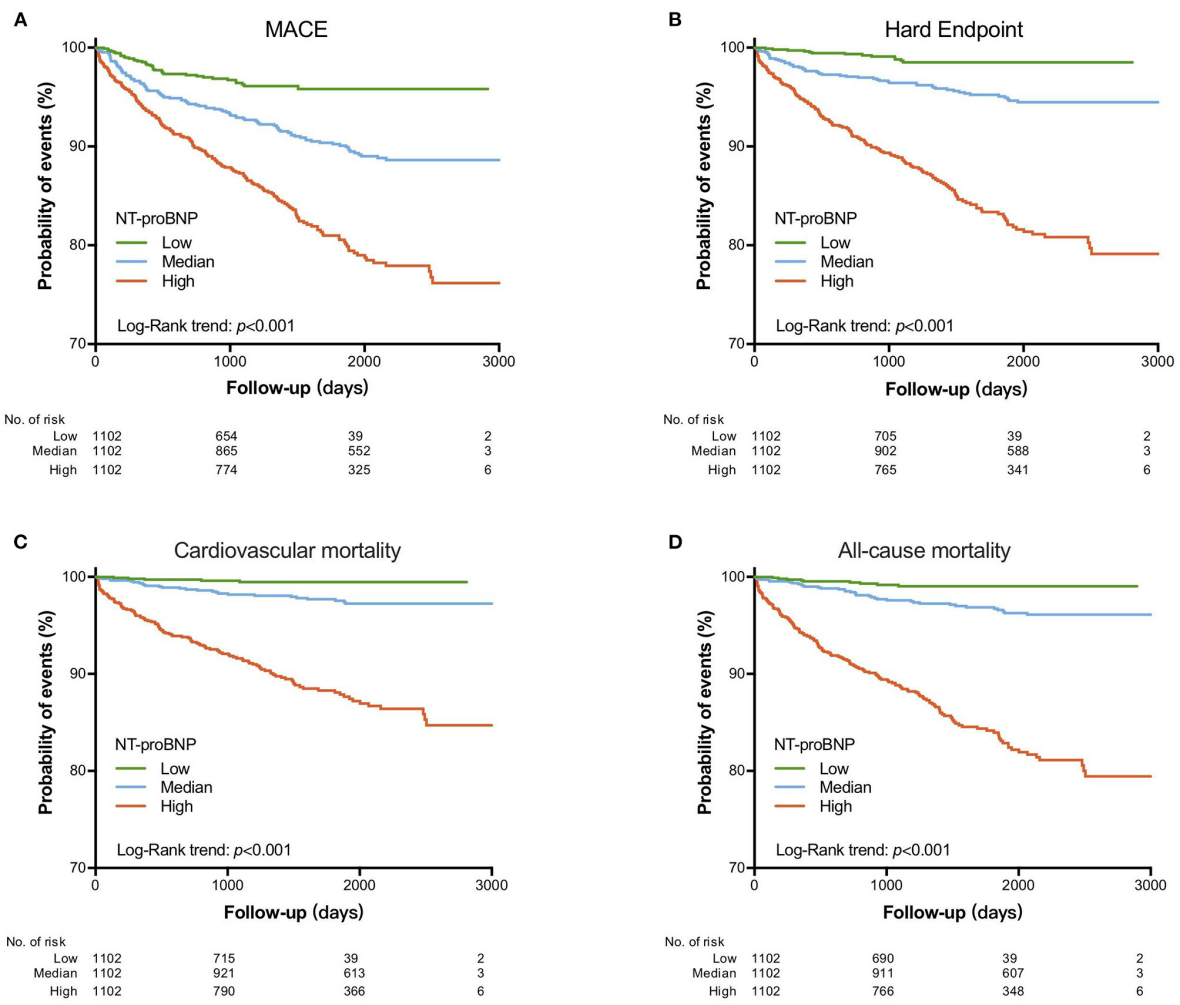
Kaplan–Meier curve for cardiovascular events according to NT-proBNP levels. **(A)** MACE; **(B)** hard endpoint; **(C)** cardiovascular mortality; **(D)** all-cause mortality.

Over an average of 4 years follow-up, 335 MACE (36 MI, 45 ischemic stroke, 103 coronary revascularization and 151 cardiac mortality) were recorded, representing 25.4 events per 1,000 person-years. As shown in Figure 1, the KM analysis showed that patients with median and high NT-proBNP values had a significantly lower cumulative MACE-free survival rates compared to those with low NT-proBNP value (Log-Rank *p* < 0.001). When hard endpoints, cardiac mortality and all-cause mortality were considered, respectively, NT-proBNP tertile 2 and 3 groups also had significantly lower event-free survival rates than tertile 1 group.

As presented in Table 2, on univariable analysis, patients in NT-proBNP tertile 2 had a 2.11-fold higher risk, while those in tertile 3 had a 4.29-fold higher risk of MACE occurrence compared with the reference group (NT-proBNP tertile 1). In Cox regression analyses with adjustment for sex and age, the HR was 3.01 (95%CI: 2.09–4.34, *p* < 0.001) for the prediction of MACE in individuals with NT-proBNP in the top tertile compared with those in the bottom tertile. After progressive adjustment for various cardiovascular risk factors including hs-CRP, NT-proBNP remained significantly associated with MACE in post-MI patients (adjusted HR: 2.99, 95%CI: 2.06–4.36). Multivariable analysis also confirmed that patients with top tertile of NT-proBNP were at an increased risk for hard endpoints (adjusted HR: 5.44; 95%CI: 2.99–9.90), cardiac mortality (adjusted HR: 5.82; 95%CI: 2.34–14.96) and all-cause mortality (adjusted HR: 5.03; 95%CI: 2.51–10.09). Additionally, we found the similar results when analyzed the association between continuous log-transformed NT-proBNP concentrations and recurrent clinical outcomes.

**Table 2:** Univariate and multivariate Cox proportional hazards regression analyses of NT-proBNP for predicting cardiovascular events.

	**Univariate analysis**		**Age- and sex-adjusted**		**Multivariate analysis**	
	**HR (95%CI)**	***p*-value**	**HR (95%CI)**	***p*-value**	**HR (95%CI)**	***p*-value**
MACE
Log (NT-proBNP)	1.70 (1.53–1.89)	<0.001	1.46 (1.31–1.64)	<0.001	1.47 (1.31–1.66)	<0.001
Low	1.00 (Reference)		1.00 (Reference)		1.00 (Reference)	
Median	2.11 (1.45–3.05)	0.001	1.94 (1.34–2.81)	0.001	1.88 (1.29–2.73)	0.001
High	4.29 (3.03–6.06)	<0.001	3.01 (2.09–4.34)	<0.001	2.99 (2.06–4.36)	<0.001
Hard endpoint
Log (NT-proBNP)	2.35 (2.06–2.69)	<0.001	1.67 (1.45–1.94)	<0.001	1.70 (1.45–1.99)	<0.001
Low	1.00 (Reference)		1.00 (Reference)		1.00 (Reference)	
Median	3.07 (1.67–5.66)	0.001	2.61 (1.42–4.81)	0.001	2.52 (1.36–4.65)	0.003
High	10.29 (6.41–16.87)	<0.001	5.55 (3.08–9.98)	<0.001	5.44 (2.99–9.90)	<0.001
Cardiac mortality
Log (NT-proBNP)	3.07 (2.59–3.64)	<0.001	1.87 (1.55–2.26)	<0.001	1.84 (1.51–2.25)	<0.001
Low	1.00 (Reference)		1.00 (Reference)		1.00 (Reference)	
Median	3.91 (1.50–9.21)	0.001	3.08 (1.18–8.05)	0.021	2.80 (1.07–7.33)	0.036
High	12.08 (5.61–19.63)	<0.001	6.99 (2.80–17.45)	<0.001	5.92 (2.34–14.96)	<0.001
All-cause mortality
Log (NT-proBNP)	2.98 (2.57–3.44)	<0.001	1.80 (1.53–2.11)	<0.001	1.74 (1.47–2.06)	<0.001
Low	1.00 (Reference)		1.00 (Reference)		1.00 (Reference)	
Median	2.94 (1.41–6.12)	0.004	2.31 (1.11–4.82)	0.025	2.24 (1.07–4.67)	0.032
High	14.91 (7.88–21.18)	<0.001	5.36 (2.69–10.70)	<0.001	5.03 (2.51–10.09)	<0.001

*MACE, major adverse cardiovascular events; NT-proBNP, N-terminal pro-brain natriuretic peptide; HR, hazard ratio; CI, confidence interval. Multivariate model is adjusted for age, sex, body mass index, family history of coronary artery disease, hypertension, smoking, diabetes, pre-revascularization, gensini score, left ventricular ejection fraction, low-density lipoprotein cholesterol, triglycerides, fasting plasma glucose, hypersensitive C-reactive protein and baseline statin use*.

To validate our findings, subgroup and sensitivity analysis were then performed. As shown in Supplementary Figure S2, a positive association between plasma NT-proBNP concentrations and incident MACE was observed in all subgroups. The significant associations between NT-proBNP and each endpoint remained unchanged in sensitivity analysis in which each of the other significant variables in univariate analysis was forced into the model with log-transformed NT-proBNP (Supplementary Table S2).

### Hs-CRP and Clinical Outcomes

Patients were divided into 2 groups according to plasma hs-CRP levels (Supplementary Table S3). Patients with elevated hs-CRP were older, had a higher prevalence of hypertension, diabetes, and cigarette smoking and STEMI, and higher levels of TC, LDL-C, apoA, apoB, FPG, HbA1c and NT-proBNP. As presented in Supplementary Table S4, univariate Cox regression models showed that the HR of recurrent MACE in post-MI patients with hs-CRP above the median value was 1.71-fold higher than ones with hs-CRP below the median level. After additional adjustment for age and sex, the relationships of incident MACE with hs-CRP remained significant. However, the relationship was no longer statistically significant after further controlling for various cardiovascular risk factors including NT-proBNP. Additionally, continuous log-transformed hs-CRP also did not contribute significantly to the prediction of recurrent clinical outcome in multivariable analysis.

### Combination of Markers

To test our assumption that the combined use of both biomarkers increases the prediction of increased risk of events, patients were divided into 6 groups according to NT-proBNP tertiles and median levels of hs-CRP: low NT-proBNP/low hs-CRP group (group 1: 704 patients); median NT-proBNP/low hs-CRP group (group 2: 599 patients); high NT-proBNP/low hs-CRP group (group 3: 350 patients); low NT-proBNP/high hs-CRP group (group 4: 398 patients); median NT-proBNP/high hs-CRP group (group 5: 503 patients); and high NT-proBNP/high hs-CRP group (group 6: 752 patients).

**Figure 2:**
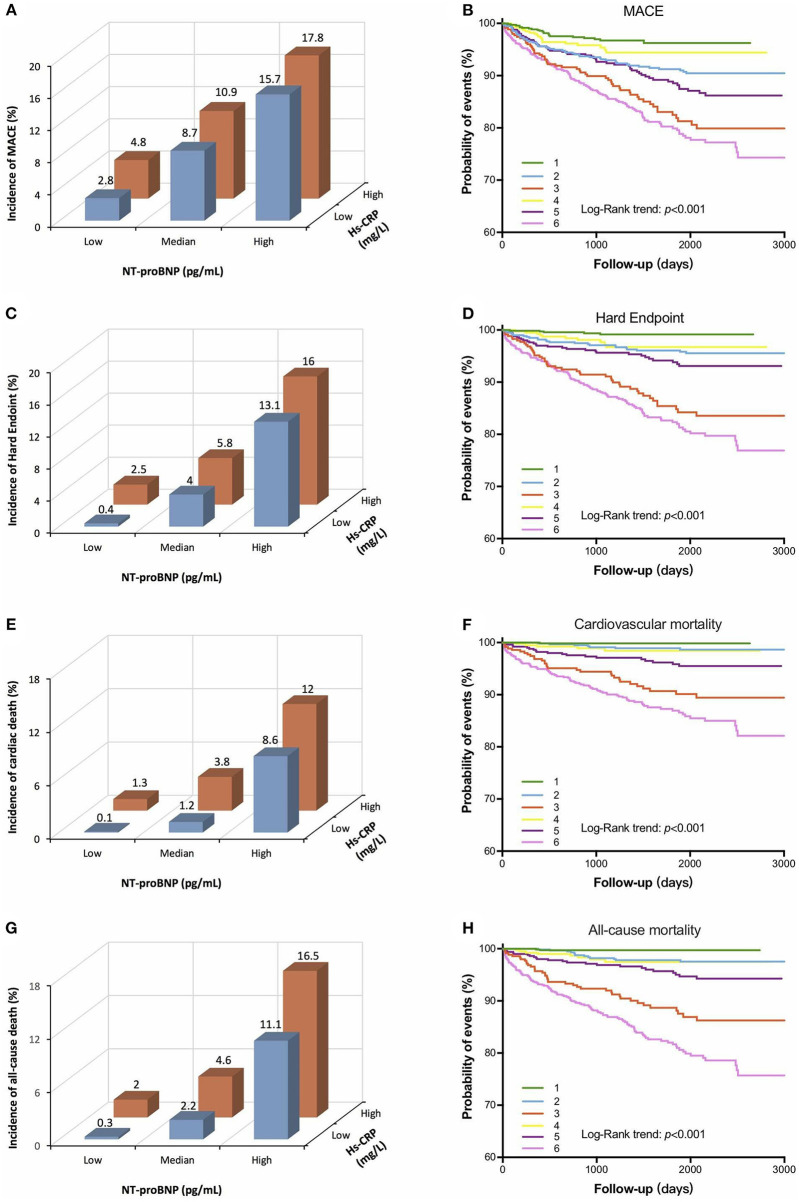
Relationship between cardiovascular markers and cardiovascular events in the follow-up period [left panel: **(A,C,E,G)**]. Kaplan-Meier curve for cardiovascular events based on the combinations of NT-proBNP and hs-CRP levels [right panel: **(B,D,F,H)**]. 1 indicates low NT-proBNP + low hs-CRP group, 2 indicates median NT-proBNP + low hs-CRP group, 3 indicates high NT-proBNP + low hs-CRP group, 4 indicates low NT-proBNP + high hs-CRP group, 5 indicates median NT-proBNP + high hs-CRP group, 6 indicates high NT-proBNP + high hs-CRP group. NT-proBNP, N-terminal pro-brain natriuretic peptide; hs-CRP, high-sensitivity C-reactive protein; MACE, major adverse cardiovascular events.

As shown in Figure 2, a very low rate of MACE was found in patients in group 1 (2.8%) and the highest 4-year rate of MACE (17.8%) was found in patients in group 6. Patients with high NT-proBNP/high hs-CRP also experienced more hard endpoints, cardiac and all-cause mortality than those with low NT-proBNP/low hsCRP. Furthermore, the KM analysis showed that patients in group 6 had significantly lower total event-free survival rates compared with those in group 1.

On multivariable Cox analysis, compared to those in group 1, those in group 6 had a more than triple risk of MACE (adjusted HR: 3.27, 95% CI: 2.02–5.29, *p* < 0.0001; Table 3). Similarly, individuals with elevated levels of NT-proBNP and hs-CRP values (group 6) had 8-fold higher risk of hard endpoints (adjusted HR: 8.14, 95% CI: 3.23-17.51, *p* < 0.0001), 16-fold higher risk of cardiac mortality (adjusted HR: 16.30, 95% CI: 2.22–27.49, *p* < 0.0001), and 11-fold higher risk of all-cause mortality (adjusted HR: 11.76, 95% CI: 2.84–28.73, *p* < 0.0001) than the patients with no elevation of either biomarker (group 1). Finally, the ability of the NT-proBNP and hs-CRP to improve risk discrimination when added to a basic cardiovascular risk factor model that included age, sex, BMI, hypertension, LDL-C, FPG, smoking status, diabetes, Gensini Score and baseline statin use was analyzed. The addition of NT-proBNP to the original model showed a significant improvement in C-statistic, NRI and IDI for each endpoint (Supplementary Table S5).

**Table 3:** Univariate and multivariate Cox proportional hazards regression analyses of the combinations of NT-proBNP and hs-CRP for predicting cardiovascular events.

	**Univariate analysis**		**Multivariate analysis**	
	**HR (95%CI)**	***p***	**HR (95%CI)**	***p***
MACE
NT-proBNP Low + Hs-CRP Low	1.00 (Reference)		1.00 (Reference)	
NT-proBNP Median + Hs-CRP Low	2.10 (1.27–3.46)	0.004	1.89 (1.14–3.13)	0.013
NT-proBNP High + Hs-CRP Low	4.28 (2.61–7.03)	<0.001	3.27 (2.02–5.29)	<0.001
NT-proBNP Low + Hs-CRP High	1.59 (0.86–2.93)	0.14	1.43 (0.77–2.65)	0.254
NT-proBNP Median + Hs-CRP High	2.81 (1.71–4.61)	<0.001	2.28 (1.38–3.77)	0.001
NT-proBNP High + Hs-CRP High	5.15 (3.28–8.09)	<0.001	3.32 (1.99–5.53)	<0.001
Hard endpoint
NT-proBNP Low + Hs-CRP Low	1.00 (Reference)		1.00 (Reference)	
NT-proBNP Median + Hs-CRP Low	4.16 (1.58–10.92)	0.004	3.44 (1.31–8.26)	0.012
NT-proBNP High + Hs-CRP Low	10.69 (4.23–19.54)	<0.001	8.14 (3.23–17.51)	<0.001
NT-proBNP Low + Hs-CRP High	3.67 (1.25–10.73)	0.018	2.94 (1.00–8.63)	0.050
NT-proBNP Median + Hs-CRP High	6.42 (2.48–16.60)	<0.001	4.36 (1.68–11.35)	0.003
NT-proBNP High + Hs-CRP High	15.21 (8.25–24.48)	<0.001	8.83 (3.45–18.62)	<0.001
Cardiac mortality
NT-proBNP Low + Hs-CRP Low	1.00 (Reference)		1.00 (Reference)	
NT-proBNP Median + Hs-CRP Low	6.02 (0.74–49.00)	0.093	4.24 (0.52–34.58)	0.178
NT-proBNP High + Hs-CRP Low	20.21 (6.84–38.50)	<0.001	15.38 (2.33–26.58)	0.005
NT-proBNP Low + Hs-CRP High	9.13 (1.07–78.13)	0.044	5.85 (0.68–50.45)	0.108
NT-proBNP Median + Hs-CRP High	20.91 (2.80–36.39)	0.003	10.79 (1.43–21.22)	0.021
NT-proBNP High + Hs-CRP High	28.89 (10.57–44.88)	<0.001	16.30 (2.22–27.49)	0.006
All-cause mortality
NT-proBNP Low + Hs-CRP Low	1.00 (Reference)		1.00 (Reference)	
NT-proBNP Median + Hs-CRP Low	5.46 (1.23–24.25)	0.026	3.80 (0.85–16.90)	0.080
NT-proBNP High + Hs-CRP Low	22.21 (7.77–36.54)	<0.001	10.25 (2.67–24.40)	0.001
NT-proBNP Low + Hs-CRP High	7.32 (1.55–34.46)	0.012	4.89 (1.03–23.15)	0.046
NT-proBNP Median + Hs-CRP High	12.38 (2.91–52.57)	0.001	6.31 (1.48–27.00)	0.013
NT-proBNP High + Hs-CRP High	29.86 (13.07–43.82)	<0.001	11.76 (2.84–28.73)	0.001

*MACE, major adverse cardiovascular events; NT-proBNP, N-terminal pro-brain natriuretic peptide; hs-CRP, high sensitivity C-reactive protein; HR, hazard ratio; CI, confidence interval. Multivariate model is adjusted for age, sex, body mass index, family history of coronary artery disease, hypertension, smoking, diabetes, pre-revascularization, gensini score, left ventricular ejection fraction, low-density lipoprotein cholesterol, triglycerides, fasting plasma glucose and baseline statin use*.

## Discussion

There is an increasing interest in identifying new biomarkers, which could be used alone or in combination in evaluating the prognosis in patients with high cardiovascular risk. We have evaluated the prognostic significance of two plasma biomarkers NT-proBNP and hs-CRP in post-MI patients. The risk for a recurrent event was significantly higher in patients with elevated NT-proBNP levels after adjustment for potential confounders. However, the independent predictivity of hs-CRP was abolished when tested in the multivariable model together with NT-proBNP. Combined use of NT-proBNP and hs-CRP increased markedly the ability to predict cardiovascular outcomes in post-MI patients, supporting the hypothesis that the additional assessment of hs-CRP leads to better risk stratification compared with NT-proBNP alone. We believe these data have potential importance for secondary prevention of CAD as well as the design of future clinical trials.

NT-proBNP, a neurohormone synthesized and released from the cardiac ventricles in response to increased wall tension, is the preferred biomarker for the detection of HF (23). However, elevated NT-proBNP concentrations may also result directly from myocardial ischemia caused by relevant coronary stenosis, even in the absence of left ventricular dysfunction (5, 6). Therefore, the potential clinical use of NT-proBNP should also be considered in a setting of CAD. A number of studies have demonstrated that elevated NT-proBNP was associated with increased risk of cardiovascular outcomes independent of LVEF in patients with stable CAD (7, 24). With respect to MI, most studies evaluated patients in the acute state of disease (25, 26), whereas data are lacking concerning the impact of NT-proBNP in patients with a history of MI. Only one prospective cohort study consisting of 983 post-MI patients showed that NT-proBNP was a powerful predictor of 1-year recurrent coronary events (24). In the present study with larger sample size and long-term follow-up period, we found that elevated NT-proBNP either as a categorical or a continuous variable was associated with a worse prognosis in post-MI patients and the association persisted after adjustment for multiple risk factors. Our results may support the potential use NT-proBNP as a helpful marker for identification of high-risk post-MI patients who may derive the greatest benefit from more aggressive pharmacologic or interventional treatment.

Inflammation plays a key role in atherosclerotic plaque progression, vulnerability, and thrombogenicity and has become an established non-traditional risk factor for adverse events (11, 15). The hs-CRP, a vascular inflammatory biomarker, have been proposed as potential indicators of underlying atherosclerotic disease (9, 14). However, the association between hs-CRP and MACE has been subject to an increasing body of research and debate. The VISTA-16 Trial suggested that a higher hs-CRP value was found to be associated with a poor prognosis after acute coronary syndromes (ACS) in 4,257 patients (13). Similar observations were also made in the TRILOGY trial that the longitudinal increases in hs-CRP were associated with increased risk of composite endpoint (27). In contrast, several studies measuring hs-CRP on presentation with MI have not identified associations with clinical outcomes (28). For example, a recent observational study enrolled 10,020 patients undergoing PCI was not able to establish an association between hs-CRP levels and cardiovascular mortality (17). In the present study, hs-CRP indeed predicted a composite endpoint in univariate analysis. However, the independent predictivity of hs-CRP was abolished when tested in the multivariable model together with NT-proBNP. Our results were in line with previous studies that once NT- proBNP was taken into account, hs-CRP did not improve predictions in patients with CAD (29, 30). Recently, Nikorowitsch et al. (19) performed a head-to-head comparison between NT-proBNP and hs-CRP in patients with CAD and found that NT-proBNP yielded additional prognostic value beyond hs-CRP. Accordingly, our findings might suggest that NT-proBNP levels may outperform hs-CRP in risk estimation.

The most impressive finding in our study is the combined effect of an increased NT-proBNP and hs-CRP on MACE. The combination of NT-proBNP and hs-CRP showed the important complementary role of hs-CRP in the prediction of cardiovascular events and further risk stratification by NT-proBNP. More precisely, patients with the similar level of NT-proBNP, those with higher level of hs-CRP are more likely to experience higher rates of cardiovascular events. Previous studies have revealed the same results that combined use of NT-proBNP and hs-CRP improves risk stratification in patients with CAD (20, 21). The increase in prognostic information provided by combined use of NT-proBNP and hs-CRP in post-MI patients may be related to the different natures of each of the two biomarkers. It is acknowledged that the activation of inflammation plays an important role not only in the pathogenesis of atherosclerosis, but also in the initiation of ACS with ensuing clinical complications (10). Elevated level of NT-proBNP, on the other hand, is released as a result of both ischemia and necrosis of myocardial cells and could predict subsequent coronary events (4, 5, 27). Besides, a strong association was found between NT-proBNP and hs-CRP, indicating close relationships between myocardial stretch and inflammatory pathways in the setting of MI (25). Thus, enhanced vascular inflammation and activation of neurohumoral axis may play synergistic roles in the process of atherosclerosis and additively predicted cardiovascular outcomes. Combined use of NT-proBNP and hs-CRP provides complementary, rather than overlapping information that increases the prediction of high risk of MACE development in post-MI patients. In brief, simultaneous assessment of these biomarkers would be more useful in predicting the MACE than the respective value of each biomarker. Moreover, this simple evaluation using a combination of biomarkers would help the clinician determine the risk stratification for cardiac events.

The important clinical implications of our study was providing insight into multiple cardiovascular biomarker prognostication and risk stratification for secondary adverse cardiovascular events in patients with prior MI as we move toward more personalized medicine. Here, we suggest post-MI patents with elevated NT-proBNP could be a target for closer follow up and potential earlier consideration of advanced therapies. This provides a foundation for future studies to further evaluate multi-marker approaches in predicting cardiovascular outcomes in patients with previous MI and develop reliable risk prediction tools.

There are several limitations of our study which need to be acknowledged. First, due to the small MI events, we did not have sufficient statistical power to analyze this outcome separately, and it was examined as part of the composite endpoints as most previous studies reported (8–10). Larger studies are needed to further evaluate the predictive risk for specific CAD endpoints. Second, despite multivariable analysis, unmeasured or residual confounding factors may not have been identified. However, this study included only patients with prior MI and analyzed the data using various models after rigorous adjustment of measured covariates. In addition, we measured these biomarkers at baseline only, so it remains unclear whether repeated measurement during the follow-up period can provide further incremental value for prediction of MACE. Moreover, HF with preserved ejection fraction is a severely underdiagnosed condition that the mean LVEF in this cohort might be theoretically caused by it in some degree. Besides, SYNTAX score is a more useful system to determine the severity of coronary arteries and provide prognostic information but it was not performed in this study. Despite these limitations, our data may serve as an important reminder to physicians that post-MI patients with both elevated NT-proBNP and hs-CRP have higher risk of recurrent MACEs and thus require more intensive treatment.

## Conclusions

In conclusion, our results demonstrate that increased plasma NT-proBNP was independently associated with recurrent MACEs in patients with prior MI and simultaneous assessment of NT-proBNP and hs-CRP conferred enhanced risk stratification beyond traditional cardiovascular risk predictors. Our study raises the possibility that the additional measurements of NT-proBNP and hs-CRP might improve risk prediction in patients with prior MI. Further studies may be needed to confirm our findings.

## Data Availability Statement

The raw data supporting the conclusions of this article will be made available by the authors, without undue reservation.

## Ethics Statement

The studies involving human participants were reviewed and approved by Fuwai Hospital Ethics Committee. The patients/participants provided their written informed consent to participate in this study.

## Author Contributions

J-JL contributed to conception and design of the study. Y-XC, SL, H-HL, and MZ organized the database. Y-XC, Y-LG, and N-QW performed the statistical analysis. Y-XC wrote the first draft of the manuscript. All authors contributed to manuscript revision, read, and approved the submitted version.

## Funding

This work was supported by the Capital Health Development Fund (201614035) and CAMS Major Collaborative Innovation Project (2016-I2M-1-011).

## Conflict of Interest

The authors declare that the research was conducted in the absence of any commercial or financial relationships that could be construed as a potential conflict of interest.

## Publisher's Note

All claims expressed in this article are solely those of the authors and do not necessarily represent those of their affiliated organizations, or those of the publisher, the editors and the reviewers. Any product that may be evaluated in this article, or claim that may be made by its manufacturer, is not guaranteed or endorsed by the publisher.
