# Supplementary material for: Prognostic Value of N-Terminal Pro-B-Type Natriuretic Peptide and High-Sensitivity C-Reactive Protein in Patients With Previous Myocardial Infarction
Source: Front Cardiovasc Med. 2022 Feb 24;9:797297. doi: 10.3389/fcvm.2022.797297 (PMC8907519; doi:10.3389/fcvm.2022.797297)

Supplementary Online Content

Table S1. Baseline clinical characteristics of study patients classified according to different NT-proBNP levels.

Variables	NT-proBNP, pg/mL			p-value
	Low (N=1102)	Median (N=1102)	High (N=1102)	
Age, y	55.6 ± 12.9	58.9 ± 13.6	68.9 ± 17.4	0.001
Male, n (%)	966 (87.7)	926 (84.0)	752 (68.2)	0.001
BMI, kg/(m^2)	24.81 ± 2.95	24.64 ± 2.79	23.51 ± 3.17	0.001
Family history of CAD, n (%)	161 (14.6)	136 (12.3)	111 (10.1)	0.005
Currently smoker, n (%)	714 (64.8)	691 (62.7)	587 (53.3)	0.002
Hypertension, n (%)	627 (56.9)	643 (58.3)	722 (65.5)	0.001
Diabetes, n (%)	351 (31.9)	357 (32.4)	453 (41.1)	0.001
Prior revascularization, n (%)	575 (52.2)	378 (34.3)	280 (25.4)	0.001
STEMI, n (%)	332 (30.1)	415 (37.7)	666 (60.4)	0.001
LVEF,%	61.94 ± 6.48	60.39 ± 6.75	57.36 ± 6.88	0.001
SBP, mmHg	124.76 ± 16.65	125.44 ± 16.79	125.64 ± 19.66	0.469
DBP, mmHg	77.63 ± 10.58	76.64 ± 10.73	72.77 ± 11.32	0.001
Gensini Score	36 (16-62)	40 (16-70)	44 (10-80)	0.001
TC, mmol/L	3.87 ± 0.99	3.98 ± 0.95	3.93 ± 1.00	0.032
HDL-C, mmol/L	1.00 ± 0.29	1.02 ± 0.27	1.03 ± 0.29	0.045
LDL-C, mmol/L	2.33 ± 0.85	2.40 ± 0.80	2.36 ± 0.84	0.380
Triglyceride, mmol/L	1.68 ± 0.92	1.66 ± 0.83	1.49 ± 0.74	0.001
ApoA, mg/dL	1.25 ± 0.26	1.30 ± 0.26	1.23 ± 0.27	0.001
ApoB, mg/dL	0.84 ± 0.27	0.88 ± 0.28	0.86 ± 0.40	0.082
Hs-CRP, mg/L	1.39 (0.74-2.84)	1.77 (0.94-3.85)	4.17 (1.56-10.91)	<0.001
FPG, mmol/L	6.01 ± 1.78	5.88 ± 1.88	6.31 ± 2.21	0.001
HBA1C, %	6.27 ± 1.10	6.48 ± 1.29	6.51 ± 1.24	0.002
Creatinine, umol/L	83.52 ± 23.00	80.73 ± 20.22	89.93 ± 29.18	0.001

Table S2. Sensitivity analysis of the association of log-transformed NT-proBNP with cardiovascular events after separate adjustment for each of the other significant variables

Adjustment Variable	MACE		Hard Endpoint		Cardiac mortality		All-cause mortality	
	**HR (95%CI)**	**p**	**HR (95%CI)**	**p**	**HR (95%CI)**	**p**	**HR (95%CI)**	**p**
Sex	1.69 (1.51-1.88)	<0.001	2.29 (1.99-2.63)	<0.001	2.97 (2.48-3.54)	<0.001	2.86 (2.46-3.34)	<0.001
Age	1.46 (1.30-1.63)	<0.001	1.67 (1.44-1.93)	<0.001	1.85 (1.54-2.23)	<0.001	1.78 (1.52-2.09)	<0.001
STEMI	1.74 (1.56-1.94)	<0.001	2.38 (2.07-2.74)	<0.001	3.10 (2.61-3.7)	<0.001	3.01 (2.59-3.50)	<0.001
LVEF	1.68 (1.51-1.87)	<0.001	2.32 (2.01-2.67)	<0.001	2.90 (2.43-3.46)	<0.001	2.82 (2.42-3.28)	<0.001
Hs-CRP	1.69 (1.52-1.88)	<0.001	2.32 (2.03-2.66)	<0.001	3.02 (2.55-3.58)	<0.001	2.93 (2.53-3.38)	<0.001

Table S3. Baseline clinical characteristics of study patients classified according to different hs-CRP levels.

Variables	Hs-CRP, mg/L		p-value
	Low (N=1653)	High (N=1653)	
Age, y	57.7 ± 14.0	64.6 ± 16.7	0.001
Male, n (%)	1407 (85.1)	1237 (74.8)	0.001
BMI, kg/(m^2)	24.43 ± 2.89	24.21 ± 3.17	0.042
Family history of CAD, n (%)	209 (12.6)	199 (12.0)	0.597
Currently smoker, n (%)	1036 (62.7)	956 (57.8)	0.004
Hypertension, n (%)	951 (57.5)	1041 (63.0)	0.001
Diabetes, n (%)	530 (32.1)	631 (38.2)	0.001
Prior revascularization, n (%)	735 (44.5)	498 (30.1)	0.001
STEMI, n (%)	505 (30.6)	908 (54.9)	0.001
LVEF,%	60.69 ± 6.97	59.11 ± 6.89	0.001
SBP, mmHg	125.28 ± 17.43	125.28 ± 18.07	0.998
DBP, mmHg	76.8 ± 10.72	74.56 ± 11.32	0.002
Gensini Score	38 (16-65)	44 (16-76)	0.059
TC, mmol/L	3.83 ± 0.95	4.02 ± 1.01	0.001
HDL-C, mmol/L	1.04 ± 0.29	0.99 ± 0.28	0.001
LDL-C, mmol/L	2.27 ± 0.81	2.46 ± 0.84	0.001
Triglyceride, mmol/L	1.60 ± 0.86	1.62 ± 0.82	0.385
ApoA, mg/dL	1.30 ± 0.26	1.22 ± 0.27	0.001
ApoB, mg/dL	0.82 ± 0.27	0.90 ± 0.37	0.001
FPG, mmol/L	5.85 ± 1.76	6.29 ± 2.15	0.001
HBA1C, %	6.3 ± 1.10	6.53 ± 1.31	0.001
NT-proBNP, pg/mL	402.0 (111.4-772.6)	775.3 (320.4-1324.9)	0.001
Creatinine, umol/L	82.65 ± 21.17	86.8 ± 27.66	0.001

Table S4. Univariate and multivariate Cox proportional hazards regression analyses of hs-CRP for predicting cardiovascular events.

	**Univariate analysis**		**Age- and sex- adjusted**		**Multivariate analysis**	
	**HR (95%CI)**	**p-value**	**HR (95%CI)**	**p-value**	**HR (95%CI)**	**p-value**
MACE						
Log (Hs-CRP)	1.24 (1.13-1.35)	<0.001	1.10 (1.00-1.20)	0.043	1.02 (0.93-1.12)	0.715
High vs. Low Hs-CRP	1.71 (1.37-2.13)	<0.001	1.33 (1.06-1.68)	0.014	1.16 (0.92-1.47)	0.216
Hard Endpoint						
Log (Hs-CRP)	1.42 (1.26-1.58)	<0.001	1.10 (0.99-1.23)	0.083	1.00 (0.90-1.13)	0.941
High vs. Low Hs-CRP	2.28 (1.73-3.01)	<0.001	1.38 (1.03-1.85)	0.030	1.18 (0.88-1.58)	0.273
Cardiac mortality						
Log (Hs-CRP)	1.64 (1.42-1.90)	<0.001	1.14 (0.98-1.31)	0.087	1.01 (0.87-1.17)	0.885
High vs. Low Hs-CRP	3.25 (2.24-4.71)	<0.001	1.55 (1.05-2.29)	0.027	1.31 (0.89-1.94)	0.173
All-cause mortality						
Log (Hs-CRP)	1.71 (1.51-1.94)	<0.001	1.20 (1.06-1.36)	0.005	1.08 (0.95-1.23)	0.258
High vs. Low Hs-CRP	3.11 (2.28-4.25)	<0.001	1.52 (1.10-2.11)	0.012	1.30 (0.93-1.80)	0.123

Table S5. Discrimination and reclassification for cardiovascular outcomes with the addition of NT-proBNP to a traditional risk factor model.

**Model**	**C-statistics (95% CI)**	**p-Value**	**NRI (95% CI)**	**p-Value**	**IDI (95% CI)**	**p-Value**
MACE						
Original mode	0.679 (0.655-0.702)					
Original mode +NT-proBNP	0.703 (0.674-0.732)	p<0.001	0.017 (0.008-0.029)	p<0.001	0.074 (0.021-0.142)	p=0.036
Original mode +hs-CRP	0.680 (0.648-0.710)	p=0.483	0.002 (0.000-0.008)	p=0.129	0.034 (-0.010-0.118)	p=0.389
Original mode+NT-proBNP-hs-CRP	0.701 (0.677-0.725)	p<0.001	0.012 (0.007-0.020)	p<0.001	0.113 (0.041-0.180)	p<0.001
Hard Endpoint						
Original mode	0.687 (0.639-0.734)					
Original mode +NT-proBNP	0.717 (0.693-0.741)	p<0.001	0.020 (0.009-0.040)	p<0.001	0.065 (0.008-0.141)	p=0.040
Original mode +hs-CRP	0.688 (0.668-0.707)	p=0.476	0.002 (0.000-0.008)	p=0.129	0.024 (-0.090-0.110)	p=0.418
Original mode+NT-proBNP-hs-CRP	0.713 (0.685-0.740)	p<0.001	0.011 (0.005-0.020)	p<0.001	0.150 (0.072-0.219)	p<0.001
Cardiac mortality						
Original mode	0.699 (0.659-0.739)					
Original mode +NT-proBNP	0.726 (0.702-0.750)	p<0.001	0.026 (0.006-0.055)	p<0.001	0.095 (0.095-0.156) 0.08	p=0.010
Original mode +hs-CRP	0.700 (0.680-0.719)	p=0.475	0.002 (0.000-0.011)	p=0.070	0.045 (-0.130-0.152)	p=0.458
Original mode+NT-proBNP-hs-CRP	0.733 (0.699-0.768)	p<0.001	0.013 (0.005-0.029)	p<0.001	0.184 (0.042-0.292)	p<0.001
All-cause mortality						
Original mode	0.689 (0.653-0.724)					
Original mode +NT-proBNP	0.720 (0.700-0.740)	p<0.001	0.030 (0.013-0.056)	p<0.001	0.082 (-0.011-0.191)	p=0.145
Original mode +hs-CRP	0.691 (0.671-0.710)	p=0.451	0.002 (0.000-0.012)	p=0.068	0.081 (-0.080-0.149)	p=0.298
Original mode+NT-proBNP-hs-CRP	0.715 (0.693-0.737)	p<0.001	0.012 (0.002-0.025)	p=0.010	0.192 (0.071-0.258)	p=0.010

NRI, net reclassification improvement; IDI, integrated discrimination improvement.
*Original Model included age, sex, body mass index, hypertension, low-density lipoprotein cholesterol, fasting blood glucose, smoking status, diabetes, Gensini Score and baseline statin use.

Figures and Legends

Figure S1. Flowchart illustrating study population.

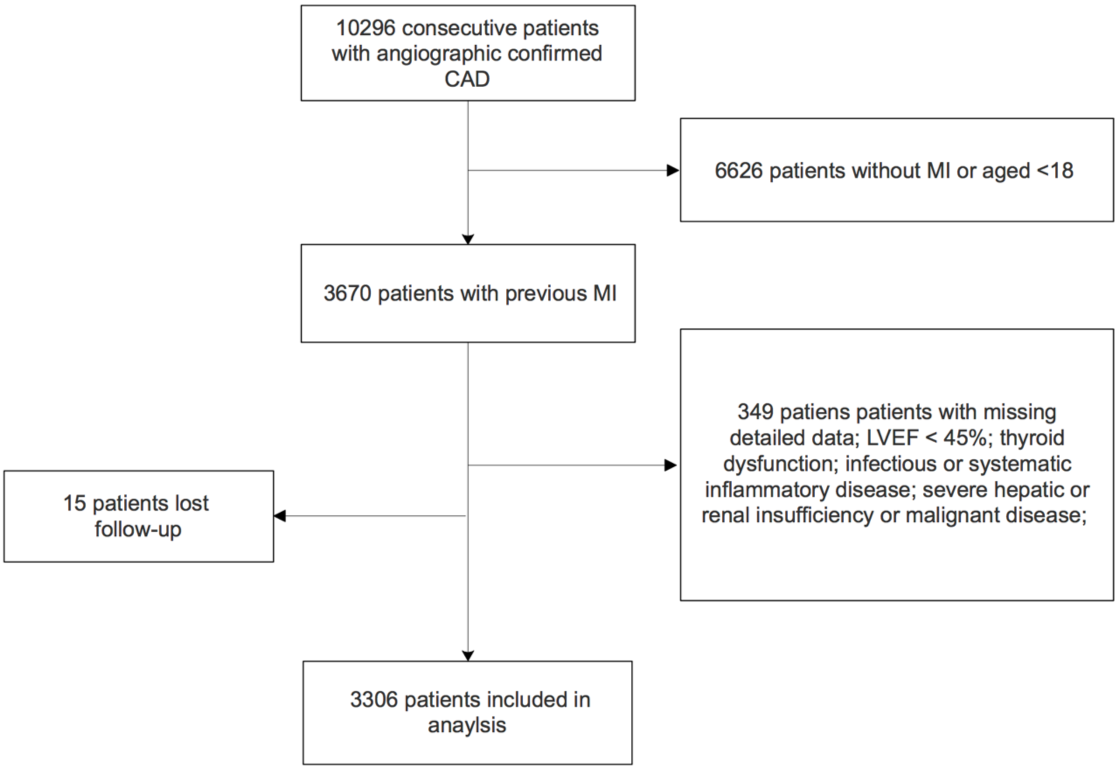

Figure S2. Subgroup analysis of the prognostic value of NT-proBNP for cardiovascular events.

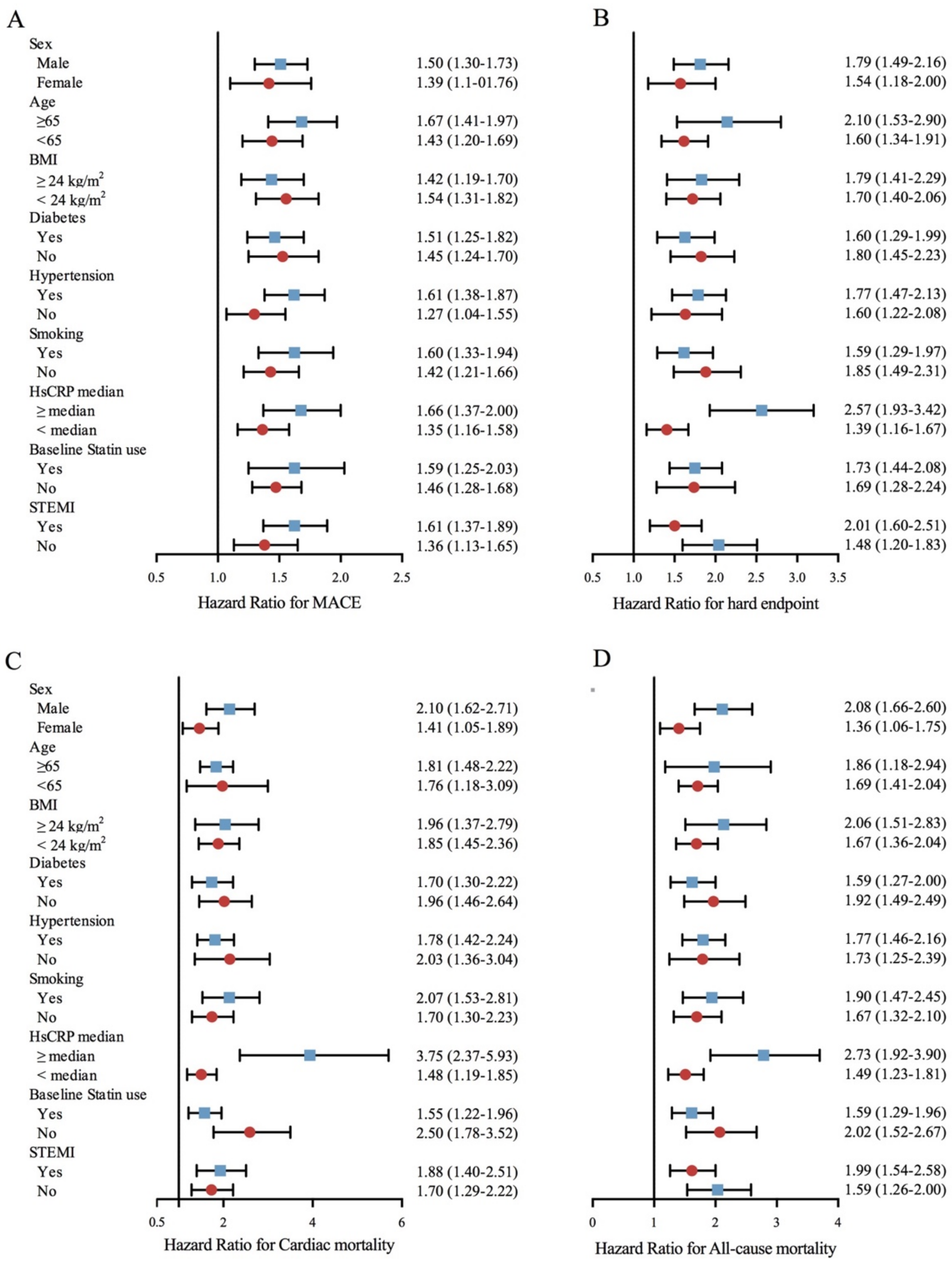

Supplement: Supplementary file 1 [file Data_Sheet_1.pdf]
